# Pegcetacoplan-induced remission in pediatric immune-complex membranoproliferative glomerulonephritis with comorbid autosomal recessive polycystic kidney disease: a case report

**DOI:** 10.3389/fmed.2026.1668375

**Published:** 2026-03-10

**Authors:** Reem Alrasheed, Abdulkarim Alanazi, Raghad Bukhari, Sawsan Albatati, Hassan Faqeehi, Saeed Alzabali

**Affiliations:** 1 Pediatric Nephrology Section, Children’s Specialized Hospital, King Fahad Medical City, Riyadh, Saudi Arabia; 2 Pathology and Laboratory Medicine, King Fahad Medical City, Riyadh, Saudi Arabia

**Keywords:** autosomal recessive polycystic kidney disease, membranoproliferative glomerulonephritis, pediatrics, pegcetacoplan, renal recovery

## Abstract

**Background:**

Membranoproliferative glomerulonephritis (MPGN) is a rare glomerular disorder characterized by immune complex or complement-mediated injury, often leading to nephrotic syndrome, hypertension, and progressive renal dysfunction. Its management remains challenging, particularly in pediatric patients with coexisting renal pathologies.

**Case presentation:**

We report a case of an 11-years-old girl who presented with nephrotic syndrome, severe hypertension, and impaired renal function. Renal biopsy confirmed immune complex-mediated MPGN (IC-MPGN), and genetic testing revealed a homozygous, pathogenic missense *PKHD1* variant (NM_138694.3:c.4870C > T; p.(Arg1624Trp), leading to a diagnosis of autosomal recessive polycystic kidney disease (ARPKD). Initial treatment with prednisolone and mycophenolate mofetil failed to halt disease progression, and the patient became dialysis dependent. Pegcetacoplan, a complement C3 inhibitor, was subsequently initiated. After 11 weeks of pegcetacoplan therapy, the patient achieved renal recovery and was successfully weaned from dialysis. Proteinuria decreased from nephrotic to sub-nephrotic levels without significant adverse effects.

**Conclusion:**

To our knowledge, this is the first pediatric case of IC-MPGN with genetically confirmed ARPKD successfully treated with pegcetacoplan. The case illustrates that renal recovery occurred following initiation of proximal complement inhibition with pegcetacoplan.

## 1 Introduction

Membranoproliferative glomerulonephritis (MPGN) is a histopathological pattern caused by glomerular injury, leading to mesangial proliferation and glomerular basement membrane thickening. While its traditional histopathological classification does not address the cause or prognosis, MPGN is now classified into immune complex MPGN (IC-MPGN) and C3 glomerulopathy (C3G) subtypes, which can be further divided into dense deposit disease (DDD) and C3 glomerulonephritis (C3GN) (1). The specific presentation varies across these subtypes; nonetheless, MPGN typically involves hematuria, proteinuria, and, in some cases, renal impairment. The prognosis is variable, depending on whether the disease is diagnosed and treated early (2). The diagnosis of MPGN is mainly based on the pattern of deposition and IF staining on biopsies (3); however, the biopsy results represent a heterogeneous group of diseases rather than distinguishing distinctive subtypes (4). Historically, the management of MPGN has relied on immunosuppressive agents; however, recent advances have introduced C3 inhibitors (e.g., pegcetacoplan) as promising therapeutic alternatives (5).

On the other hand, autosomal recessive polycystic kidney disease (ARPKD) is a genetic disorder that causes cysts to replace the renal parenchyma, eventually leading to renal impairment. Disease processes involving the gastrointestinal tract cause liver fibrosis and portal hypertension alongside typical nephrological manifestations, including enlarged kidneys, hypertension, and chronic renal impairment (6). ARPKD is a genetic condition that is not known to be associated with autoimmune or glomerular diseases.

In this paper, we present the case of a pediatric patient with MPGN who was incidentally found to have ARPKD through genetic testing. She received the novel therapy pegcetacoplan, which ameliorated the pathological presentation of MPGN despite the co-occurrence of ARPKD.

## 2 Case description

An 11-years-old Saudi female patient, born to consanguineous parents (first-degree relatives), with no remarkable family history of liver disease, presented with symptoms of a viral upper respiratory tract infection. She had a known history of hypertension for 5 months prior to presentation, which had not been investigated. Additionally, she had undergone an upper endoscopy 5 years earlier due to episodes of vomiting, which showed normal findings. At the time of her current assessment, she was found to have severe hypertension, hepatosplenomegaly, and significant ascites. Her hypertension was managed with carvedilol, a calcium channel blocker, and furosemide.

A laboratory investigation revealed a hemoglobin level of 13.2 g/L a normal WBC count, and a platelet count of 131 × 10^9^. Additional lab tests revealed hypoalbuminemia with an albumin level of 22 g/L, nephrotic-range proteinuria, and hematuria. Her urea concentration was 10 mmol/L and creatinine concentration was 66 μmol/L, indicating renal function impairment. Complement testing revealed a low C3 level of 0.6 g/L (normal value: 0.81–1.57 g/L) and a normal C4 level of 0.14 g/L (normal value: 0.129–0.392 g/L), while C3 nephritic factor (C3NeF) was negative. Hepatitis B, hepatitis C, and HIV tests were all negative. Autoimmune marker tests, including ANA, anti-dsDNA, ANCAs, and anti-GBM, were negative.

Renal imaging at presentation revealed bilaterally enlarged echogenic kidneys (measuring 12 and 13 cm, respectively) with loss of corticomedullary differentiation. Hepatic ultrasound demonstrated heterogeneous liver echotexture without intrahepatic or extrahepatic biliary dilatation. The common bile duct measured 0.2 cm. The gallbladder appeared contracted and was difficult to evaluate. The pancreas appeared normal. The patient had splenomegaly (13.3 cm), and the portal and hepatic veins were patent. Liver function tests, including ALT, AST, and bilirubin, were within normal limits. To further support the diagnosis, endoscopy revealed esophageal varices. Her ascites worsened, requiring pigtail drainage. Collectively, the presence of portal hypertension with preserved liver biochemistry and ultrasound findings without biliary dilatation was considered most consistent with congenital hepatic fibrosis related to ARPKD. Congenital hepatic fibrosis was not confirmed histologically, as liver biopsy was not performed.

Thus, genetic testing was performed via a next-generation sequencing panel for nephropathy-related genes at a certified reference laboratory. The analysis identified a homozygous missense pathogenic variant in the *PKHD1* gene: NM_138694.3:c.4870C > T, resulting in an amino acid change p.(Arg1624Trp). This variant, also listed under SNP ID rs200391019, is classified as pathogenic (class 1) based on ACMG guidelines and reported in ClinVar in association with ARPKD. *In silico* analysis supported pathogenicity (SIFT: deleterious; MutationTaster: polymorphism; PolyPhen: not reported). The variant is extremely rare, with a gnomAD allele frequency of 0.00015. The patient was confirmed to be homozygous for this variant, which is consistent with the clinical and radiological diagnosis of ARPKD. No pathogenic variants in complement regulatory genes associated with MPGN were identified on the tested panel, although this does not exclude complement dysregulation not captured by the assay.

**FIGURE 1:**
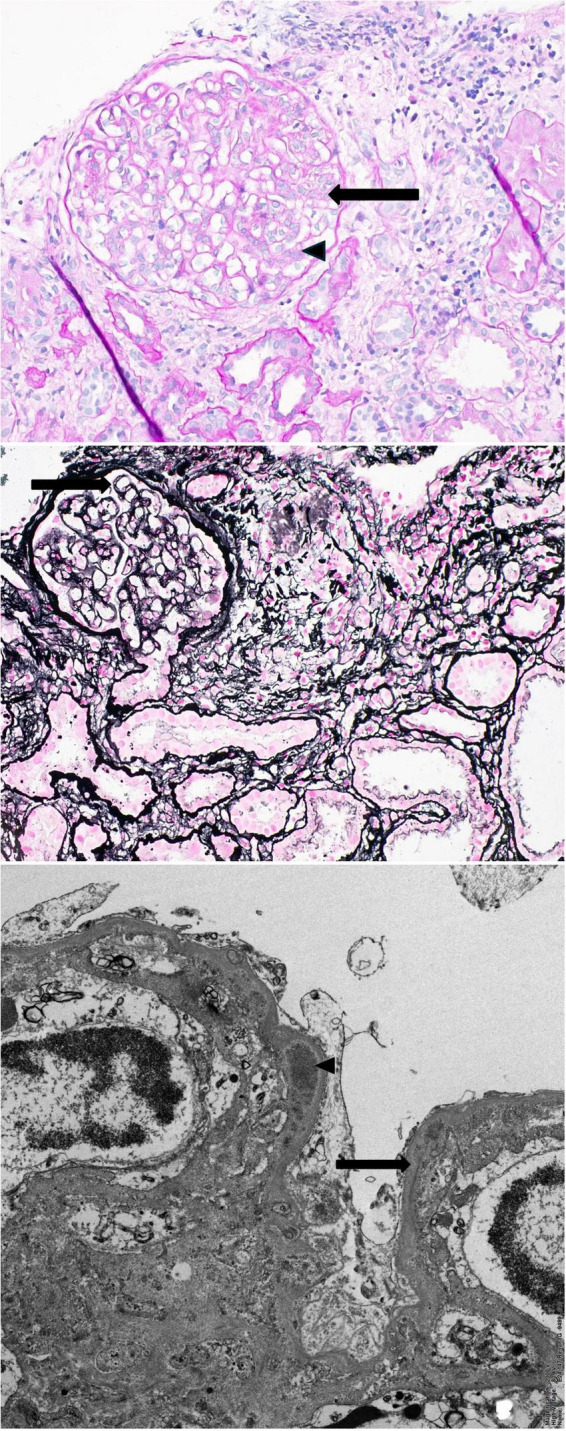
Renal biopsy findings consistent with immune complex-mediated membranoproliferative glomerulonephritis (IC-MPGN). Upper: Light microscopy (PAS stain, ×400); the glomerulus shows segmental endocapillary hypercellularity (arrow) and mesangial matrix expansion and hypercellularity (arrow head); Middle: The glomerulus is showing thickening of the glomerular basement membrane and segmental double countor (arrow); Lower: Ultrastructural image demonstrates glomerular basement membrane with double contour (arrow), associated with intramembranous electron dense deposits (arrow head).

A percutaneous kidney biopsy was performed in September 2024 and demonstrated a membranoproliferative pattern of glomerular injury. Light microscopy showed lobulated glomeruli with mesangial expansion and endocapillary hypercellularity, with features of basement membrane remodeling (Figure 1). Electron microscopy showed electron-dense deposits with glomerular basement membrane duplication, supporting a diagnosis of immune complex–mediated MPGN (Figure 1). Immunofluorescence (three glomeruli) demonstrated granular mesangial and capillary wall staining for IgG (1+) and C3 (1+), with no staining for IgA, IgM, C1q, fibrinogen, or kappa and lambda light chains, and no extraglomerular staining. Although isolated hypocomplementemia raised the differential diagnosis of C3 glomerulopathy, the immunofluorescence pattern was not C3-dominant.

Following the biopsy, the patient was prescribed prednisone (60 mg/m^2^/day) and mycophenolate mofetil, titrated to 1.2 g/m^2^/day. Despite treatment, her condition deteriorated with worsening ascites, anasarca, severe hypertension, and declining renal function. Her creatinine continued to rise. Serial complement testing revealed persistently low serum C3 levels (0.681 g/L), with persistent nephrotic-range proteinuria. She developed a clinical picture of rapidly progressive glomerulonephritis (RPGN). High-dose intravenous methylprednisolone (10 mg/kg/day) was administered for 5 days, followed by oral prednisone at 60 mg/m^2^/day. The patient showed temporary improvement, with increased urine output, and was discharged.

Two weeks later, she returned with recurrence of edema, ascites, and uncontrolled hypertension. Her creatinine fluctuated between 120 and 400 μmol/L over the following month, and proteinuria persisted at 6 g/day. Despite repeated pulse steroids and maintenance immunosuppression with mycophenolate mofetil, renal function continued to decline, and she developed oliguria and diuretic-resistant volume overload. Renal replacement therapy was initiated in the form of intermittent hemodialysis for diuretic-resistant volume overload in the setting of oliguria and progressive azotemia.

Given the refractory nature of her disease and evidence of complement activation, pegcetacoplan, a complement C3 inhibitor, was initiated at a dose of 1080 mg subcutaneously twice weekly (2 months after the biopsy). Prior to treatment, the patient received meningococcal vaccination and prophylactic antibiotics according to safety protocols. Concomitant immunosuppression was continued at the time of pegcetacoplan initiation. Mycophenolate mofetil was maintained at 750 mg twice daily. Prednisolone was continued initially and then tapered gradually over approximately 5 months.

**FIGURE 2:**
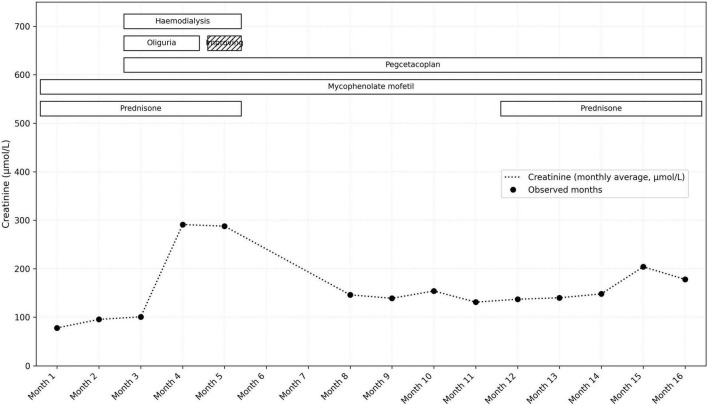
The change in serum creatinine over time and correlation with treatment interventions.

**FIGURE 3:**
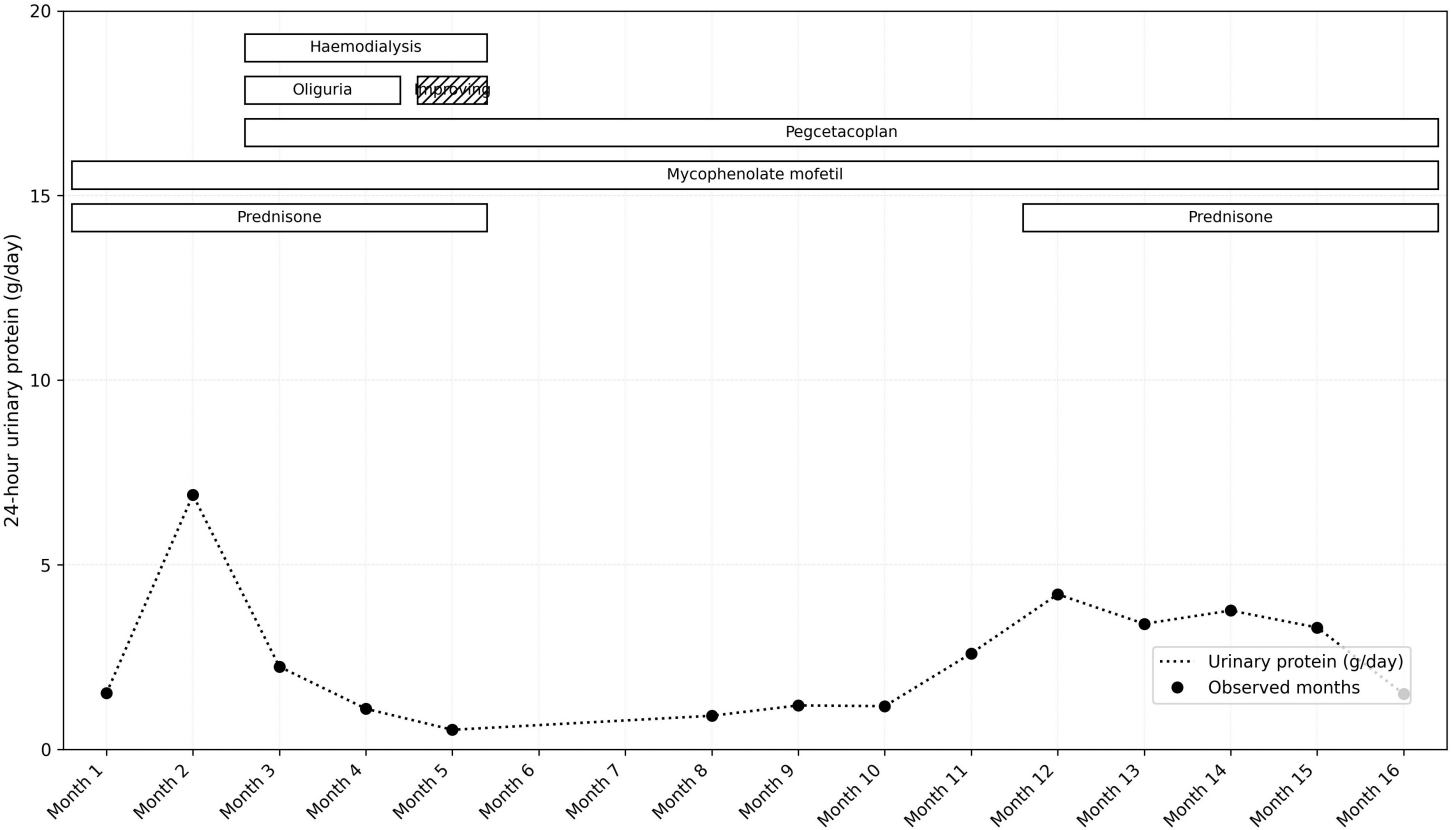
The change in 24-h urinary protein measurements in g/day over time and correlation with treatment interventions.

At the time of pegcetacoplan initiation, the patient was severely nephrotic, with persistent proteinuria of 6 g/day and hypoalbuminemia (serum albumin 24 g/L). Following treatment, proteinuria gradually improved, decreasing to 0.9 g/day by week 25, while serum albumin increased to 35 g/L. By week 9 of pegcetacoplan treatment, the patient began producing urine, her blood pressure improved, and dialysis was discontinued after 11 weeks. On follow-up, creatinine stabilized at 140 μmol/L (Figure 2), and proteinuria had stabilized at 0.9 g/day, consistent with stage 3 chronic kidney disease (Figure 3).

Twenty weeks after starting pegcetacoplan, follow-up renal ultrasound showed a reduction in kidney size to 9–9.5 cm bilaterally, persistent increased echogenicity, and continued loss of corticomedullary differentiation, along with features of liver disease and splenomegaly, further supporting the diagnosis of ARPKD. Serial complement testing revealed a gradual rise, reaching normal levels by January 2025 (1.372 g/L), and peaking above normal (2.307 g/L) by April 2025, which coincided with clinical recovery. C4 remained consistently within normal range. The pattern of isolated C3 consumption, along with C3 deposition on immunofluorescence and the presence of dense deposits on electron microscopy, was strongly suggestive of complement dysregulation and alternative pathway activation. Although immunofluorescence images of the kidney biopsy were unavailable due to technical issues, the histologic findings were consistent with complement-mediated membranoproliferative glomerulonephritis.

**FIGURE 4:**
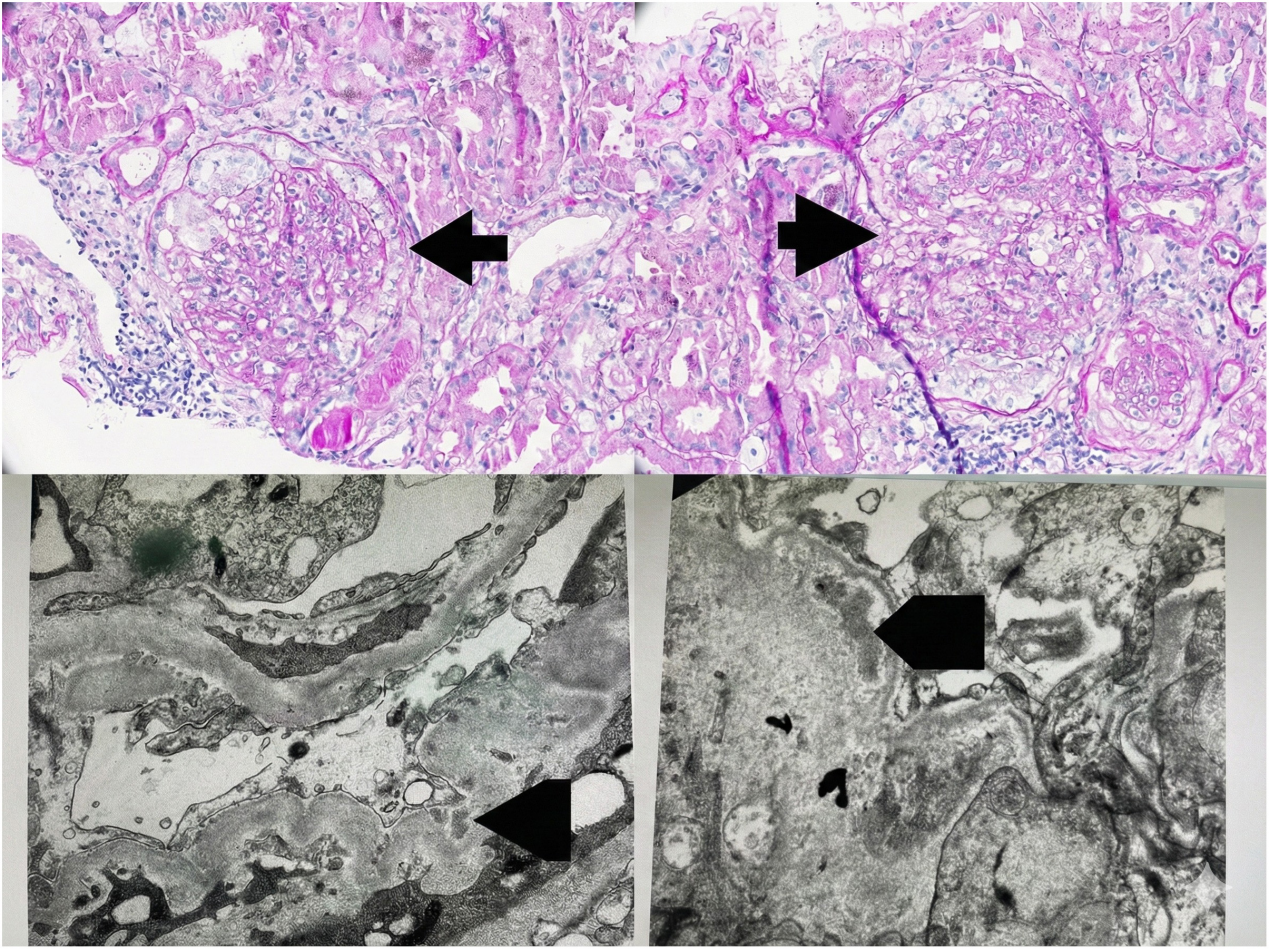
Second renal biopsy findings consistent with immune complex-mediated membranoproliferative glomerulonephritis (IC-MPGN). Top panels (LM): Representative glomeruli showing a membranoproliferative pattern, with lobular accentuation/mesangial expansion and capillary wall thickening with duplication (“double contour” change) (arrows). Bottom panels (EM): Ultrastructural examination demonstrates intramembranous electron-dense deposits within the glomerular basement membrane (GBM) (arrowheads), accompanied by diffuse podocyte foot process effacement, consistent with an immune complex–mediated glomerular injury pattern.

**FIGURE 5:**
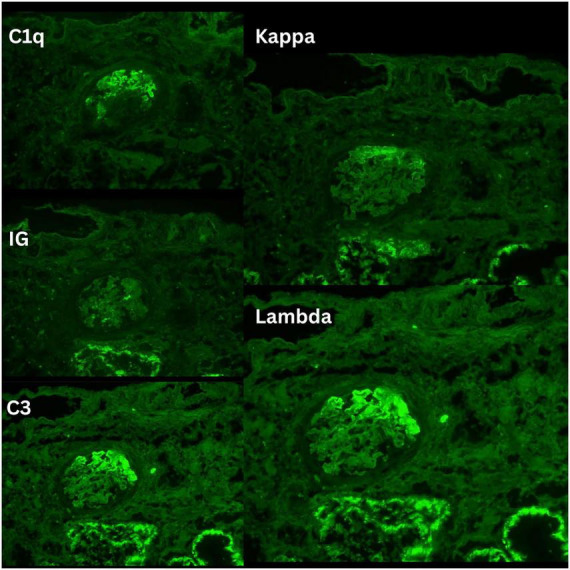
Immunofluorescence findings on the second renal biopsy. Representative immunofluorescence photomicrographs demonstrate granular mesangial and segmental capillary wall staining within glomeruli. Staining is shown for C3, immunoglobulin (Ig; labeled “IG”), and C1q, with accompanying kappa and weaker/trace lambda light-chain reactivity. The overall pattern supports immune complex deposition with complement activation.

Over time, as her clinical status improved, all antihypertensive and diuretic medications were gradually tapered. By 25 weeks post-treatment, she no longer required any medications for blood pressure or fluid management. After approximately 1 year of treatment (September 2025), the patient developed worsening proteinuria, reaching 4 g/day, and a serum creatinine of 148 μmol/L (Figures 2, 3), prompting a repeat kidney biopsy, which demonstrated crescentic activity (Figure 4). Immunofluorescence demonstrated segmental granular mesangial and capillary wall staining for IgG (2+), C3 (trace), C1q (2+), kappa (2+), and lambda (trace) light chains. No staining was observed for IgA, IgM, or fibrinogen. No extraglomerular staining was identified (Figure 5).

She received pulse methylprednisolone, followed by oral prednisolone, and mycophenolate mofetil was continued. Proteinuria improved again after giving the second course of steroids at 1.5 g/day. At the time of writing, she remains on prednisolone with a gradual taper.

There were no major side effects, breakthrough infections, or bleeding events during therapy; however, an elevated activated partial thromboplastin time (APTT) was noted.

## 3 Discussion

The above-discussed case highlights an unusual overlap of two distinct kidney pathologies, ARPKD and MPGN. To our knowledge, this is the first case report of an ARPKD patient who developed idiopathic immune complex glomerulonephritis.

The comorbidity of these conditions poses a significant challenge to diagnosis and therapy, as each condition is associated with significant morbidity. The patient’s initial presentation of significant nephrosis, hypertension, and a mildly decreased complement level is not typical of ARPKD. Importantly, ARPKD is associated with an insidious decline in kidney function and subnephrotic-range proteinuria (7). Alternatively, the patient’s acute presentation suggested a glomerular kidney pathology, with possible alternative complement pathway activation. Indeed, kidney biopsy confirmed an immune complex process, immune complex-mediated membranoproliferative glomerulonephritis. Nonetheless, subsequent genetic tests revealed a mutation that caused ARPKD, providing explanations for hepatosplenomegaly, portal hypertension, and nephromegaly on ultrasonography. An additional consideration is the viral upper respiratory tract infection preceding presentation. Intercurrent infections are recognized precipitants of complement activation and may unmask or exacerbate complement-mediated glomerular injury, potentially acting as a “trigger” for clinical onset or flare in susceptible individuals. In this case, the temporal association supports a possible contributory role; however, a causal relationship cannot be established.

Furthermore, we believe that the combination of MPGN and ARPKD accelerated the decline in her kidney function. While ARPKD alone can lead to end-stage renal disease (ESRD) in childhood, this decline typically occurs over several years; in this case, the acute glomerular inflammation caused by a swift decline over several weeks. While both ARPKD and IC-MPGN involve renal injury processes that may secondarily affect complement activity, any potential relationship remains speculative and unproven; their concurrence in this patient is likely coincidental. ARPKD is not known to cause glomerulonephritis, and further research is needed to support these speculations. In this case, the diagnosis of MPGN was driven by glomerular changes on light microscopy and electron microscopy together with the immunofluorescence profile and is therefore unlikely to represent a biopsy artifact attributable to cystic kidney disease. There are few reports of adult patients with a confirmed diagnosis of PKD and comorbid glomerulonephritis (particularly SLE or IgA nephropathy). Still, there are no reports of MPGN in a patient with ARPKD (8).

Another possible consideration is that ARPKD has been associated with low-grade inflammatory activity and complement pathway engagement. Experimental data in rodent models show upregulation of complement components and the presence of activated C3 fragments (iC3) in cyst fluid and urine. These findings suggest that complement activity may accompany cystic kidney disease, although a direct pathogenic role has not been established (9).

Based on the current understanding of the field, ARPKD and MPGN are two distinct entities from a genetic and etiological perspective, and most likely, these two diseases occurred independently in this case. Nonetheless, these coinciding pathologies could point toward a “second hit phenomenon” as chronically inflamed kidneys, which also have reduced mass, lead to a lower threshold for glomerulonephritis. In addition, the co-occurrence of both pathologies in one patient caused a double burden on her kidneys; although complement inhibitors could immunologically control MPGN, the presence of ARPKD limited renal recovery.

Evidence has strongly supported the use of complement inhibitors in the treatment of MPGN, including pediatric cases. Moreover, in a recent phase III trial (VALIANT) involving patients with C3 and immune complex MPGN, pegcetacoplan led to a 68% mean reduction in proteinuria by 26 weeks compared with placebo. In addition, pegcetacoplan has been shown to significantly stabilize renal function and improve the eGFR within a period of 6 months of treatment. One striking finding is that 71% of those patients treated with pegcetacoplan achieve clearance of C3 deposition, as opposed to 0% in the placebo group (5). Our patient’s course aligns with these findings, as she experienced a reduction in proteinuria and partial recovery of renal function, possibly limited by coexisting ARPKD; nonetheless, reports of such findings are rare in pediatric MPGN patients. Together with those from the literature, our findings suggest that early initiation of complement inhibitors halts the progression in renal impairment and results in recovery of renal function. Notably, another case report of a 9-years-old male who had MPGN revealed that the condition was successfully managed by pegcetacoplan (10). Similarly, a case series of children from Europe who were treated with off label pegcetacoplan for 12 weeks revealed a reduction in proteinuria, normalization of complement levels, and improved or at least stabilized eGFRs in the majority of the children. Similar to our patient, 3 of those patients had impaired kidney function at the beginning of the treatment course but showed improvement in the eGFR after 3 months of therapy (11). However, those patients had acute impairment of their renal function, unlike our patient, who underwent a period of dialysis dependency. Previous reports of pediatric cases with MPGN mirror adult case presentations. Our case, however, extends beyond these findings by demonstrating the chance for renal recovery, even after a patient undergoes a period of dialysis dependency, when proximal complement inhibition is introduced earlier in the course of the kidney disease. Notably, it is likely that without complement inhibition, MPGN would have led to irreversible damage in our patient and thus permanent dialysis dependence; instead, attenuation of ongoing complement dysregulation has allowed their residual nephrons to recover.

In terms of the effects, only an elevated coagulation profile was observed when she was an outpatient, and there were no serious infections or medical issues during her treatment. The elevated coagulation profile could be attributed to the artificially increased coagulation profile with the treatment; however, continued monitoring is needed.

A limitation of this report is that the complement evaluation was incomplete, which limits the mechanistic interpretation of the observed response to C3 inhibition. Although C3NeF was negative and no pathogenic complement regulatory gene variants were identified on the available nephropathy gene panel, factor H autoantibody testing was not performed, and broader complement functional/expanded genetic assessment was not available. These gaps may affect the interpretation of the driver of complement activation and the basis for pegcetacoplan responsiveness. An additional limitation is the lack of immunofluorescence images in the first biopsy due to archival technical issues. This constraint may limit histopathologic confirmation of complement dominance. However, the diagnosis was supported by the pattern of glomerular injury on light and electron microscopy, together with the serologic profile and clinical evolution.

In conclusion, this case report describes the diagnosis and treatment of an 11-years-old patient with a histological diagnosis of MPGN and a genetic diagnosis of ARPKD. She was dependent on dialysis for a while, and after treatment with anti-C3 therapy, she was weaned off dialysis, and her CKD was downgraded to stage 3. This case illustrates that early intervention and early complement inhibition can alter the disease course of MPGN in pediatric patients. Importantly, the combination of intensive immunotherapy and a proximal complement inhibitor (pegcetacoplan) resulted in halting the progression of renal impairment to end-stage disease. While this temporal association is encouraging, a direct causal relationship cannot be confirmed based on a single case, and spontaneous or delayed recovery remains possible. Future studies are needed to support the long-term safety and efficacy of proximal complement inhibitors.

## Data Availability

The original contributions presented in this study are included in this article/supplementary material, further inquiries can be directed to the corresponding author.

## References

[1] Sethi S Nester CM Smith RJH. Membranoproliferative glomerulonephritis and C3 glomerulopathy: resolving the confusion. *Kidney Int.* (2012). 81:434–41. 10.1038/ki.2011.399 22157657 PMC4428602

[2] Servais A Noël LH Roumenina LT Le Quintrec M Ngo S Dragon-Durey MA et al. Acquired and genetic complement abnormalities play a critical role in dense deposit disease and other C3 glomerulopathies. *Kidney Int.* (2012) 82:454–64. 10.1038/ki.2012.63 22456601

[3] Vivarelli M van de Kar N Labbadia R Diomedi-Camassei F Thurman JM. A clinical approach to children with C3 glomerulopathy. *Pediatr Nephrol.* (2022) 37:521–35. 10.1007/s00467-021-05088-7 34002292

[4] Noris M Daina E Remuzzi G. Membranoproliferative glomerulonephritis: no longer the same disease and may need very different treatment. *Nephrol Dial Transplant.* (2023) 38:283–90. 10.1093/ndt/gfab281 34596686

[5] Nester CM Bomback AS Ariceta Iraola MG Delmas Y Dixon BP Gale DP et al. VALIANT: a randomized, multicenter, double-blind, placebo (PBO)-controlled, phase 3 trial of pegcetacoplan for patients with native or post-transplant recurrent glomerulopathy (C3G) or primary immune complex membranoproliferative glomerulonephritis (IC-MPGN). *J Am Soc Nephrol.* (2024) 35:1. 10.1681/ASN.2024qdwvz5bg 38015564

[6] Rossetti S Harris PC. Genotype-phenotype correlations in autosomal dominant and autosomal recessive polycystic kidney disease. *J Am Soc Nephrol* (2007) 18:1374–80. 10.1681/ASN.2007010125 17429049

[7] Hartung EA Guay-Woodford LM. Autosomal recessive polycystic kidney disease: a hepatorenal fibrocystic disorder with pleiotropic effects. *Pediatrics.* (2014) 134:e833–45. 10.1542/peds.2013-3646 25113295 PMC4143997

[8] D’Cruz S Singh R Mohan H Kaur R Minz RW Kapoor V et al. Autosomal dominant polycystic kidney disease with diffuse proliferative glomerulonephritis - An unusual association: A case report and review of the literature. *J Med Case Rep.* (2010) 4:125. 10.1186/1752-1947-4-125 20429898 PMC2873454

[9] Li X. *Polycystic Kidney Disease | Exon Publications.* (2015). Available online at: https://exonpublications.com/index.php/exon/issue/view/4 (accessed July 14, 2025). 27512748

[10] Guzman GL Perry KW. Pegcetacoplan for the treatment of paediatric C3 glomerulonephritis: a case report. *Nephrology.* (2025) 30:e70001. 10.1111/nep.70001 39871447 PMC11772912

[11] Mancuso MC Cugno M Griffini S Grovetti E Nittoli T Mastrangelo A et al. Efficacy of complement inhibition with pegcetacoplan in children with C3 glomerulopathy. *Pediatr Nephrol.* (2025) 40:1959–63. 10.1007/s00467-025-06673-w 39841237

